# Evaluation of the effect of multipoint intra-mucosal vaginal injection of a specific cross-linked hyaluronic acid for vulvovaginal atrophy: a prospective bi-centric pilot study

**DOI:** 10.1186/s12905-021-01435-w

**Published:** 2021-08-28

**Authors:** Nicolas Berreni, Jennifer Salerno, Thierry Chevalier, Sandrine Alonso, Pierre Mares

**Affiliations:** 1 Gynecology Private Practice, Perpignan, France; 2 Carémeau University Hospital, Nimes, France; 3 BESPIM (Biostatistics, Clinical Epidemiology, Public Health, Innovation and Methodology Laboratory), Carémeau University Hospital, Nimes, France

**Keywords:** Collagen, Desirial, Dryness, Dyspareunia, FSFI, Hyaluronic acid, Menopause, PGI-I, Vulvo-vaginal atrophy

## Abstract

**Background:**

Vulvo-vaginal atrophy (VVA) is one of the common consequences of estrogen deficiency especially after the menopause. Several studies have assessed the effects of Hyaluronic acid (HA) on physical and sexual symptoms associated with VVA with promising results. However, most of these studies have focused on subjective assessment of symptom response to topically administered preparations. Nonetheless, HA is an endogenous molecule and it is logical that its effects are best realized if injected in the superficial epithelial layers. Desirial® is the first crosslinked HA that is administered by injection in the vaginal mucosa. The aim of this study was to explore the effect of multipoint vaginal intra-mucosal injections of specific cross-linked hyaluronic acid (DESIRIAL®, Laboratoires VIVACY) on several clinical and patient reported core outcomes.

**Methods:**

A cohort bi-centric pilot study. The chosen outcomes included change in vaginal mucosa thickness, biological markers for collagen formation, vaginal flora, vaginal pH, vaginal health index, vulvo-vaginal atrophy symptoms and sexual function 8 weeks post Desirial® injection. Patients’ satisfaction was also assessed using the patient global impression of improvement (PGI-I) scale.

**Results:**

A total of 20 participants were recruited between 19/06/2017 and 05/07/2018. At the end of the study, there was no difference in the median total thickness of the vaginal mucosa or in procollagen I, III or Ki67 fluorescence. However, there was a statistically significant increase in COL1A1 and COL3A1 gene expression (*p* = 0.0002 and *p* = 0.0010 respectively). There was also a significant reduction in reported dyspareunia, vaginal dryness, vulvar pruritus, vaginal chafing and significant improvement in all female sexual function index dimensions. Based on PGI-I, 19 patients (95%) reported varying degrees of improvement where, 4 (20%) felt slightly better; 7 (35%) better and 8 (40%) much better.

**Conclusions:**

Multi-point vaginal intra-mucosal injections, of Desirial® (a crosslinked HA) was significantly associated with the expression of CoL1A1 and CoL3A1 suggesting stimulation of collagen formation. Furthermore, there was a significant reduction in VVA symptomatology and a significant improvement in patient satisfaction and sexual function scores. However, there was no demonstrable change in the total vaginal mucosal thickness.

*Study registration *ID-RCB: 2016-A00124-47, Protocol code number: LOCAL/2016/PM-001.

**Supplementary Information:**

The online version contains supplementary material available at 10.1186/s12905-021-01435-w.

## Background

Vulvo-vaginal atrophy (VVA) is one of the common consequences of estrogen deficiency especially after the menopause [1–4]. Several clinical syndromes are associated with VVA, including, dryness, irritation, pruritus, dyspareunia, and recurrent urinary tract infections, which can have a significant negative impact on the woman’s quality of life [5]. However, the onset of these can be subtle and gradual and start to become noticeable after other menopausal symptoms have subsided. It is reported that up to 55%, 41% and 15% of postmenopausal women suffer from vaginal dryness, dyspareunia and recurrent urinary tract infections respectively [6–9]. Nevertheless, it is suggested that the actual prevalence of these problems is higher but the majority of women do not seek medical help for their symptoms [6].

The mainstay of VVA management is symptomatic and includes lifestyle modifications, non-hormonal (e.g. vaginal lubricants or moisturizers and laser treatments) and hormonal treatment options. Vaginal lubricants are, primarily, used to relieve vaginal dryness during intercourse, and hence, do not provide an effective solution for the chronic and complex nature of VVA symptomatology. In contrast, vaginal moisturizers are “bio-adhesive” products that facilitate water retention have been reported to improve vaginal irritation and dyspareunia when used regularly [10]. Nonetheless, this was not associated with improvement in the overall vaginal epithelium maturation index [11]. In recent years, there have been several claims for the use of radiofrequency and laser for the treatment of vaginal menopausal symptoms [12–15]. Nevertheless, the FDA has issued an alert to patients stressing that the use of such procedures may be associated with serious adverse events and that the safety and effectiveness of energy-based devices for the treatment of these conditions has not been established [16]. Evidence from meta-analyses of several randomized studies support the effectiveness of hormonal treatments, both local and systematic, in relieving VVA associated symptoms [17–19]. However, a limited number of studies have assessed the sustained effect of such treatments beyond 6 months of therapy. Moreover, their contraindications and personal choice are limiting factors to the widespread and long-term use of these treatment options. Therefore, at present, there is still a need for a safe and effective solution for the management of VVA related symptoms.

Hyaluronic acid (HA) is an extracellular matrix key molecule present in several tissues including the vaginal mucosa. It is a polysaccharide from the glycosamino-glycan family that plays a major role in maintaining water balance and the regulation of inflammation, immune response, scarring and angiogenesis [20, 21]. Synthetic HA preparations are available in the form of local gels and have a “medical device” status. Several studies have assessed the effects of HA on physical and sexual symptoms associated with VVA with promising results [22–25]. However, most of these studies have focused on subjective assessment of symptom response to topically administered preparations. Nonetheless, HA is an endogenous molecule and it is logical that its effects are best realized if injected in the superficial epithelial layers. Desirial® is the first crosslinked HA that is administered by injection in the vaginal mucosa.

## Methods

The aim of this prospective bi-centric pilot study was to explore the effect of multipoint vaginal intra-mucosal injections of specific cross-linked hyaluronic acid (DESIRIAL®, Laboratoires VIVACY) on several clinical and patient reported core outcomes and assess the feasibility of evaluating these outcomes. The comprehensive set of chosen outcomes for this study included the change in vaginal mucosa thickness, biological markers for tissue regeneration, vaginal flora, vaginal pH and vaginal health index 8 weeks post Desirial® injection. We measured several patient-reported outcomes including change in sexual function and reported rates of VVA related symptoms over the same time point. Patients’ satisfaction was assessed using the patient global impression of improvement (PGI-I) scale at the end of the study.

### Participants

The study population comprised of postmenopausal women (between 2 and 10 after the menopause) referred to the menopause clinic with symptoms of vaginal discomfort and/or dyspareunia secondary to vaginal dryness. Women had to be ≥ 18 years and < 70 years of age with a BMI of < 35. Participants were recruited from one of 2 participating units (Centre Hospitalier Régional Universitaire, Nîmes (CHRU), France and Karis Medical Center (KMC), Perpignan, France). Women were considered eligible if they were affiliated to or were beneficiaries of a health insurance plan and they knew they would be available for the 8-week planned follow-up period. Women participating in other studies at the time were not eligible for recruitment. The presence of ≥ stage 2 apical pelvic organ prolapse, stress urinary incontinence, vaginismus, vulvovaginal or urinary tract infection, Hemorrhagic or neoplasic genital pathologies, hormone-dependent tumor, genital bleeding of unknown etiology, recurrent porphyria, uncontrolled epilepsy, heart conduction disorders, recurrent angina, rheumatic fever, previous vulvovaginal or uro-gynecological surgery, hemostatic disorders and tendency to develop hypertrophic scarring were considered criteria for exclusion. Women on anti-hypertensive, steroidal and non-steroidal anti-inflammatory drugs, anticoagulants, major antidepressants or aspirin and those known to have hypersensitivity to HA, mannitol, betadine, lidocaine, amide-linked local anesthetics or to any of the excipients in the lidocaine-based anesthetics were not considered eligible for the study.

### Baseline assessment: Day zero (D0)

At baseline, women were asked to complete a female sexual function index (FSFI) [26] and information relating to VA symptoms (Dyspareunia, vaginal dryness, vaginal chaffing and vulvar pruritus) were collected using 0–10 visual analogue scales (VAS). Pre-intervention assessment included a check for vaginal pH, a clinical evaluation of the vagina using the Bachmann Vaginal Health index (VHI) [27], a pap smear to assess vaginal flora and a vaginal mucosal biopsy. Vaginal pH was measured near the planed injection site and in the lateral vaginal fornix. For the vaginal flora, the Nugent score [28, 29] which provides a tool to quantify the vaginal ecosystem where scores of 0–3, 4–6 and 7–10 represent normal flora, intermediate flora and vaginosis respectively. All vaginal flora assessments were performed at the Bacteriology department, CHRU, Nimes. A standardized procedure was used for taking vaginal mucosal biopsies. A 6–8 mm punch biopsy was taken from the area of the planned injection site. Mucosal biopsies were histologically assessed for the thicknesses of the basal, intermediate and superficial layers. Biopsies were also used to measure the COL1A1 and COL3A1 mRNA using RT-PCR and procollagen I and III immune-histo fluorescence as surrogates for collagen expression and the fluorescence of the proliferation marker Ki67 as a surrogate for the mucosal mitotic activity. Genetic tests were carried out by BioAlternatives laboratory, 1bis rue des Plantes, 86160 GENCAY, France (Protocol available on request).

### Intervention

**Fig. 1:**
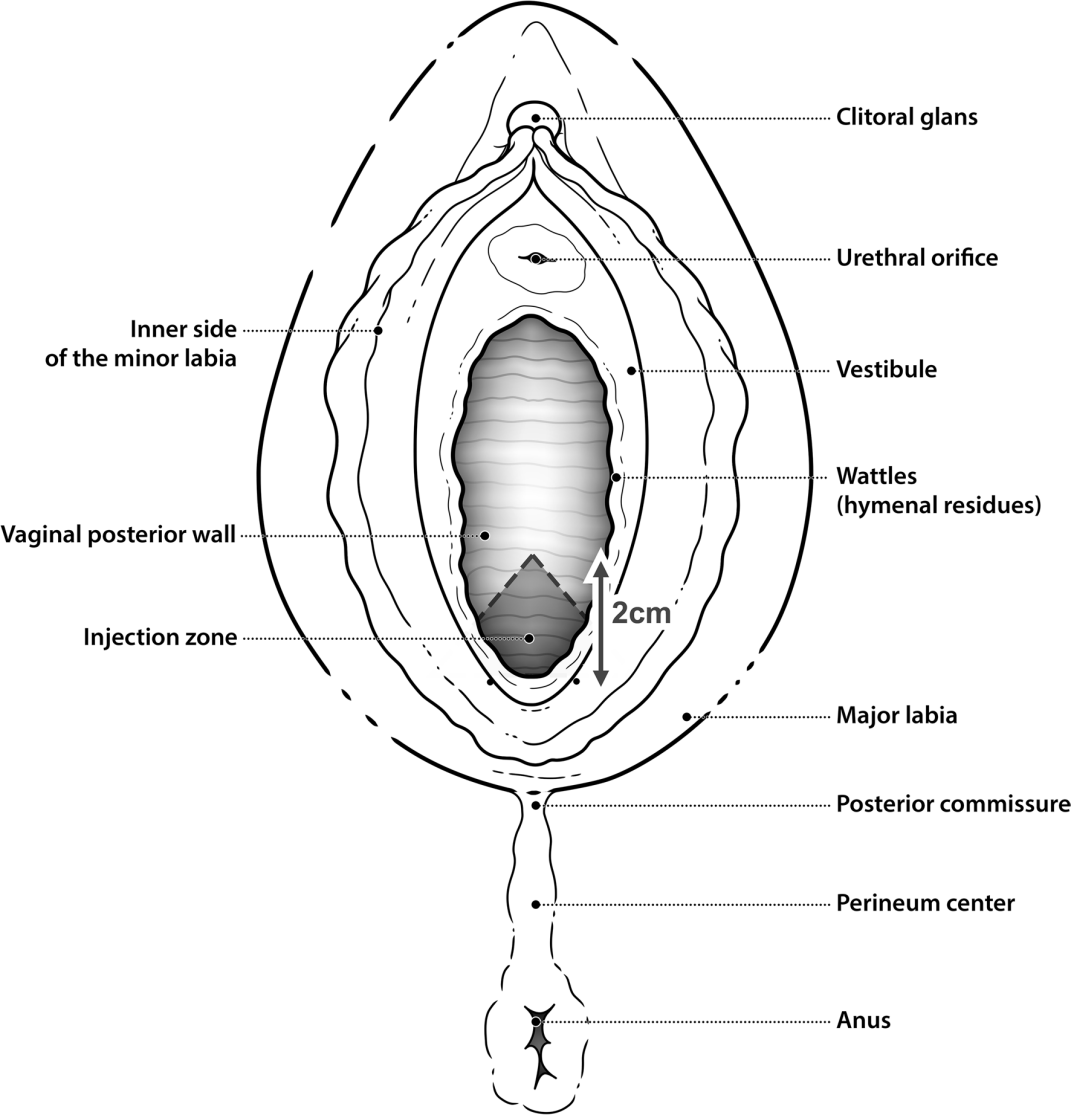
Diagram demonstrating Desirial® injection sites

Once the baseline samples and measures were complete, the crosslinked HA (Desirial®) was injected by one of 2 trained specialists following the standard protocol. Desirial® [NaHa (sodium hyaluronate) crosslinked IPN-Like 19 mg/g + Mannitol (antioxidant)] is an injectable HA gel of non-animal origin intended for single use and packaged in pre-filled syringes (2 × 1 ml). It is a class III medical devise (CE 0499) intended for intra-mucosal injection in women for the bio-stimulation and rehydration of the superficial layers of the mucous membrane of the genital areas (Laboratoires Vivacy, 252 rue Douglas Engelbart—Archamps Technopole, 74160 ARCHAMPS, France). About ten injections, 70–100 µl each (0.5–1 ml in total), were carried in 3–4 horizontal lines over a triangular area on the posterior vaginal wall with its base at the level of the fourchette and the apex 2 cm above (Fig. 1).

### End of study assessment: week 8 (W8)

The end of study assessment was scheduled 8 weeks after inclusion. Women were assessed for the same parameters assessed at baseline. Additionally, patients were asked to complete a global impression of improvement (PGI-I) satisfaction scale [30].

### Statistical analysis

In view of the absence of prior data and the pilot nature of the study, it was not possible to perform a formal a priori sample size calculation. Hence a convenience sample size of 20 patients, in total, was chosen based on the capacities of the two participating units and sufficient to obtain reasonable estimates of the proposed outcome criteria. Statistical analysis was performed using SAS software (9.4; SAS Inc., Cary NC) with a significance level set at 5%. Changes at 8 weeks were tested using the Wilcoxon signed-rank test for continuous variables and McNemar’s test for categorical variables.

### Ethics

The study was approved by Comité d’ethique du CHU Carémeau de Nimes (ID-RCB: 2016-A00124-47, Protocol code number: LOCAL/2016/PM-001). All study participants signed a valid written consent. Patients were compensated up to € 200 for the 2 study visits and the 2 biopsies.

## Results

A total of 20 participants were recruited between 19/06/2017 and 05/07/2018 (8 patients from CHRU and 12 patients from KMC). There were no protocol violations to the set a priori inclusion / exclusion criteria. All injection procedures were uneventful and were performed within 20 min. Demographic and baseline characteristics of study participants are presented in Table 1. At baseline there were 12 (60%) of the 20 women using treatments for their symptoms (6 hormonal and 6 non-hormonal) while at W8 2 patients (10%) only were still such treatments (*p* = 0.002).

**Table 1:** Participants demographics and presenting symptoms

Parameter	Mean	SD
*Demographics*		
Age	58	5.75
Weight (Kg)	59	10.63
Height (cm)	161.3	5.89
BMI	22.64	3.73
Age at menopause	50.7	4.46
Menopause years	7.9	4.53

Parameter	Number	Percentage
*Presenting symptoms*		
Dryness	16	80
Vaginal Chaffing	10	50
Vulvar Pruritus	7	35
Dyspareunia	16	80
Recurrent urinary tract infections	3	15
Other (lateral vaginal pain)	1	5

The results of the clinical and patient-reported outcomes are presented in Tables 2 and 3. One patient declined the W8 vaginal biopsy; hence complete data for histological and genetic analysis was available for 19/20 participants. There was no difference in the median total thickness of the vaginal mucosa at W8 compared to D0, however, there was increase in the median basal layer thickness from 70.28 to 83.25 μm but this increase was not statistically significant (*p* = 0.8596). There was no statistically significant difference in procollagen I, III or Ki67 fluorescence before and after treatment. Nonetheless, there was a statistically significant increase in COL1A1 and COL3A1 gene expression (*p* = 0.0002 and *p* = 0.0010 respectively). There was no statistically significant change but a trend in favor of improving the vaginal flora post Desirial® injection (n = 11, *p* = 0.1250). Similarly there was a trend to a reduction in vaginal pH, both at the vicinity of injection (n = 17) sites and in the lateral vaginal fornicies (n = 19), but this difference was not statistically significant (*p* = *p* = 0.0574 and 0.0955 respectively) (Table 2).

**Table 2:** Clinical outcomes at W8 compared to D0

Outcome	D0	W8	*p* value
*Vaginal mucosa thickness (N = 19)*			
Superficial			
Median	28.41	26.36	*p* = 1
IQR	17.31; 38.78	21.5; 35.89	
Range	10.13; 116.88	10.6; 102.12	
Intermediate			
Median	114.4	112.5	*p* = 0.6507
IQR	85.58; 141.74	71.76; 162.12	
Range	40.6; 230.53	36.19; 237.26	
Basal			
Median	70.28	83.25	*p* = 0.8596
IQR	59.1; 103.79	63.78; 89.77	
Range	36.61; 128.23	36.69; 116.96	
Total			
Median	213.65	212.33	*p* = 0.5949
IQR	139.33; 300.76	175.09; 261.58	
Range	97.66; 439.7	86.65; 456.33	
*Vaginal Flora (N = 11)*			
Nugent score			
Median	5	5	*p* = 0.5469
IQR	3; 7	3; 5	
Range	2; 8	1; 7	
*Vaginal pH*			
Vicinity of the injected area (N17)			
Median	6	5.5	*p* = 0.0574
IQR	5.5; 6.5	5; 6	
Range	5; 7.5	4.5; 7	
Lateral vaginal pouch (N = 19)			
Median	6	5.5	p = 0.0955
IQR	5.5; 7	4.5; 6.5	
Range	4; 7.5	4.5; 7.5	
*Bachman VHI (N = 18)*			
VHI			
Median	12	21	*p* < 0.0001
IQR	10; 16	17; 22	
Range	5; 17	12; 25	
*Procollagen I and III proteins mean fluorescence intensity/μm*^*2*^* (N = 19)*			
Procollagen I mean fluorescence intensity/μm^2^			
Median	47.87	61.91	*p* = 0.9843
IQR	35; 72.71	27.3; 82.78	
Range	16.06; 146.87	8.01; 145	
Procollagen III mean fluorescence intensity/μm^2^			
Median	11.99	10.49	*p* = 0.7086
IQR	3.32; 27.36	4.6; 20.71	
Range	0.27; 72.27	0.7; 60.13	
*COL1A1 and COL3A1 gene expression N = 19*			
COL1A1/GAPDH			
Median	0.0035	0.0168	*p* = 0.0002
IQR	0.0014; 0.0053	0.0091; 0.0403	
Range	0.0006; 0.0151	0.0012; 0.1178	
COL3A1/GAPDH			
Median	0.0003	0.0009	*p* = 0.0010
IQR	0.0002; 0.0005	0.0004; 0.0025	
Range	0.0001; 0.0012	0.0001; 0.0068	
*Mitotic activity of the mucosa (Ki67) N = 19*			
Ki67 positive nucleus mean/mm^2^			
Median	229.4	195.09	*p* = 0.8288
IQR	114.54; 426.16	76.76; 463.05	
Range	5.4; 695.26	0; 668.75

**Table 3:** Patient-reported outcomes at W8 compared to D0

Outcome	D0	W8	*p* value
*Pain/dyspareunia VAS (N20)*			
VAS			
Median	7.25	0	*p* = 0.0001
IQR	3.5; 8.25	0; 3.25	
Range	0; 10	0; 10	
*Number of women reporting Dyspareunia (N = 20)*			
Suffer with Dyspareunia	16	8	*p* = 0.0078
*Number of women reporting Vaginal dryness (N = 20)*			
Suffer with dryness	16	2	*p* = 0.0005
*Number of women reporting Vaginal Chaffing (N = 20)*			
Suffer with chaffing	10	2	*p* = 0.0215
*Number of women reporting Vulvar pruritus (N = 20)*			
Suffering with pruritus	7	1	*p* = 0.0313
*Female sexual function index (FSFI) scores*			
Full scale			
Median	18.8	24.65	*p* = 0.0019
IQR	15.7; 21.9	19.9; 28.7	
Range	2; 28.8	4.8; 34.5	
Desire domain			
Median	3	3.6	*p* = 0.0010
IQR	1.8; 3.6	2.7; 4.2	
Range	1.2; 4.2	1.8; 5.4	
Arousal domain			
Median	3	4.2	*p* = 0.0549
IQR	1.5; 4.5	2.55; 4.8	
Range	0; 5.1	0; 5.7	
Lubrication domain			
Median	2.4	4.65	*p* = 0.0005
IQR	0.6; 3	3.75; 5.1	
Range	0; 6	0; 6	
Orgasm domain			
Median	3.8	4.8	*p* = 0.3515
IQR	0.6; 5.6	1.8; 5.6	
Range	0; 6	0; 6	
Satisfaction domain			
Median	4	5.2	*p* = 0.0126
IQR	2.8; 4.8	4; 5.6	
Range	0.8; 6	1.2; 6	
Pain domain*			
Median	1.6	3.6	*p* = 0.0005
IQR	0; 3.6	1.2; 5.4	
Range	0; 4.8	0; 6	

^*^A higher score indicates less pain

Patient-reported outcomes were available for all study participants. Based on PGI-I, One participant (5%) reported no change post-injection while the remaining 19 patients (95%) reported varying degrees of improvement where, 4 (20%) felt slightly better; 7 (35%) better and 8 (40%) much better. There was also a significant reduction in reported dyspareunia, vaginal dryness, vulvar pruritus, vaginal chafing and the FSFI total score, as well as its desire, lubrication, satisfaction and pain dimensions (Table 3).

## Discussion

### Summary and analysis of findings

The hypothesis underpinning this study was that multi-point Desirial® injections, in the posterior vaginal wall would lead to thickening of the vaginal mucosa, lowering of the vaginal pH, improve vaginal flora, induce collagen formation and improve VA symptomatology. We were able to demonstrate significant improvement in all patient reported outcomes including dyspareunia, vaginal dryness, vaginal chaffing and vulvar pruritus. There was also significant improvement in the VHI, FSFI and a significant reduction in the number of women needing to use alternative treatments to control their symptoms. Of relevance, it was feasible to collect information on all the outcomes determined at the outset and to be able to deliver the intervention for all the study participants. Moreover, 75% of the study participants reported that their symptoms were better or much better at the end of the study period.

However, despite a slight increase in the mean thickness of the basal layer, we were not able to demonstrate a significant impact on the total thickness of the vaginal mucosa. Although our study was not powered to assess the effectiveness of Desirial® on improving vaginal mucosa thickness, we believe the outcome is of relevance in light of the statistically significant increase in the expression of the CoL1A1 and CoL3A1 markers at W8 compared to D0, which signify collagen stimulation. However, there are some issues that need to be taken into account prior to considering its use in future studies. Firstly, is the 8-week follow-up period too short to demonstrate an improvement in the total thickness of the mucosa? It is possible that the changes identified in the basal layer might have been realized in the other layers if the follow-up period was longer. Secondly, is the histological thickness of the mucosal layers a reflection of tissue regeneration? Histological assessment of vaginal mucosal thickness does not necessarily take into account the basal lamina, which includes the regenerative tissue in contact with the underlying connective tissue.

### Strengths and limitations

We appreciate that the small number of participants and the lack of a priori formal sample size are limitations to our study; nonetheless, both are standard features of a pilot study. It is for this reason that we avoided extending our findings to claims of clinical effectiveness or ineffectiveness. However, one of the main strengths to our work is that it enabled us to generate data for several outcomes that will assist us in calculating formal samples sizes for future definitive studies. Moreover, the pilot allowed us to test our recruitment strategy, attrition rates, feasibility of sample collection and outcomes analysis, which will inform any further related work. Finally, the spectrum of outcomes we evaluated that includes objective clinical outcomes, biomarkers and patient reported outcomes assessed using validated measures, are major strengths to our study.

### Comparison to other studies

Desirial® is the first crosslinked HA that is administered by injection in the vaginal mucosa. For it to be delivered through this route it is essential that the product is fluid enough to be easily injected into specialized and dense connective tissue, while maintaining its hygroscopic properties. This is achieved by optimizing the size of the gel molecule and level of gel crosslinkage ensuring high gel concentration while maintaining low viscosity and elasticity.

The beneficial effects of HA has been assessed in several studies most of which were non-inferiority RCTs that have compared HA to other forms of treatment, mainly hormonal [22–25]. The HA in these studies was topically administered. HA is an endogenous molecule characterized by an extremely important capacity to fix and transport water. With advancing age, the endogenous HA quantity of the vaginal mucosa decreases sharply, as well as, its thickness and vascularization and this consequently reduces plasma transudation and lubrication. In this study we have demonstrated that Desirial® injections were associated with significant improvement in all VVA associated symptoms. These findings concur with a previous study that was undertaken by Berreni et al. as part of the regulatory approvals of Desirial® (Unpublished – supplementary information) (Additional file 1). Although only speculative, it is plausible that this improvement is secondary to the restoration of the plasma transfer to the superficial layers of the vaginal epithelium.

Crosslinked HA gel has also been shown to increase type I collagen and elastin synthesis and therefore the thickness of the surrounding tissues [31, 32]. In our study we did no demonstrate a significant difference in the fluorescence of procollagen I and III after treatment. Nonetheless, there was a statistically significant increase in COL1A1 and COL3A1 gene expression. Therefore, it is possible that Desirial® might have a stimulatory effect on collagen formation in the vaginal, however, larger studies with longer follow-up will be required to conform or refute this possibility.

### Impact on practice and future research

The present study has provided baseline data and potential effect sizes for several outcomes that will help in future sample size calculations. Moreover, the study has demonstrated the feasibility of collecting different outcomes. However, it has also highlighted several issues that will need careful consideration when planning future research in this field. Although Desirial® seems to significantly improve VVA symptoms and sexual function, the mechanistic pathway(s) by which it works is unclear. There seems to be preliminary evidence that it stimulates collagen formation as identified from the significant expression of CoL1A1 and CoL3A1. Nonetheless, similar effects were not realized for procollagen 1, procollagen 3 and Ki67. Therefore it is imperative that additional histological and biological markers are explored in future studies.

## Conclusion

Multi-point vaginal intra-mucosal injections, of Desirial® (a crosslinked HA) was significantly associated with the expression of CoL1A1 and CoL3A1 suggesting stimulation of collagen formation, a significant reduction in VVA symptomatology, as well as the use of alternative treatments. Additionally, there was a significant improvement in patient satisfaction and sexual function based on PGI-I and FSFI scores respectively. However, there was no demonstrable change in the total vaginal mucosal thickness.

## Supplementary Information

**Additional file 1**. A poster summary of an unpublished study undertaken for DESIRIAL regulatory approvals.

## Data Availability

The datasets used and/or analysed during the current study are available from the corresponding author on reasonable request.

## Abbreviations

CHRU: Centre Hospitalier Régional Universitaire, Nîmes
FSFI: Female sexual function index
HA: Hyaluronic acid
KMC: Karis Medical Center
PGI-I: Patient global impression of improvement scale
VAS: Visual analogue scales
VHI: Vaginal Health index
VVA: Vulvo-vaginal atrophy

## References

[1] Raz R, Stamm WE (1993). A controlled trial of intravaginal estriol in postmenopausal women with recurrent urinary tract infections. N Engl J Med.

[2] Griebling TL, Nygaard IE (1997). The role of estrogen replacement therapy in the management of urinary incontinence and urinary tract infection in postmenopausal women. Endocrinol Metab Clin North Am.

[3] Smith P, Heimer G, Norgren A, Ulmsten U (1990). Steroid hormone receptors in pelvic muscles and ligaments in women. Gynecol Obstet Invest.

[4] Kalogeraki A, Tamiolakis D, Relakis K, Karvelas K, Froudarakis G, Hassan E (1996). Cigarette smoking and vaginal atrophy in postmenopausal women. Vivo (Brooklyn).

[5] Woods NF (2012). An overview of chronic vaginal atrophy and options for symptom management. Nurs Womens Health.

[6] van Geelen JM, van de Weijer PHM, Arnolds HT (2000). Urogenital symptoms and resulting discomfort in non-institutionalized Dutch women aged 50–75 years. Int Urogynecol J.

[7] Stenberg Å, Heimer G, Ulmsten U, Cnattingius S (1996). Prevalence of genitourinary and other climacteric symptoms in 61-year-old women. Maturitas.

[8] Utian WH, Schiff I. NAMS-gallup survey on women’s knowledge, information sources, and attitudes to menopause and hormone replacement therapy. Menopause. 1994. 10.1097/GME.0000000000001213 30358709

[9] Foxman B (1999). Urinary tract infection in postmenopausal women. Curr Infect Dis Rep.

[10] Nachtigall LE (1994). Comparative study: Replens* versus local estrogen in menopausal women†. Fertil Steril.

[11] van der Laak JAWM, de Bie LMT, de Leeuw H, de Wilde PCM, Hanselaar AGJM (2002). The effect of Replens(R) on vaginal cytology in the treatment of postmenopausal atrophy: cytomorphology versus computerised cytometry. J Clin Pathol.

[12] González Isaza P, Jaguszewska K, Cardona JL, Lukaszuk M (2018). Long-term effect of thermoablative fractional CO2 laser treatment as a novel approach to urinary incontinence management in women with genitourinary syndrome of menopause. Int Urogynecol J.

[13] Gaviria JE, Lanz JA. Laser Vaginal Tightening (LVT)—evaluation of a novel noninvasive laser treatment for vaginal relaxation syndrome. J Laser Heal Acad Artic J LAHA. 2012.

[14] Gaspar A, Addamo G, Brandi H. Vaginal fractional CO2 laser: a minimally invasive option for vaginal rejuvenation. Am J Cosmet Surg. 2011.

[15] Salvatore S, Leone Roberti Maggiore U, Origoni M, Parma M, Quaranta L, Sileo F (2014). Microablative fractional CO2 laser improves dyspareunia related to vulvovaginal atrophy: a pilot study. J Endometr..

[16] Smith J. FDA warning shines light on vaginal rejuvenation. ObGynNews. 2018;1–5.

[17] Suckling JA, Kennedy R, Lethaby A, Roberts H. Local oestrogen for vaginal atrophy in postmenopausal women. In: Suckling JA, editor. Cochrane database of systematic reviews. Chichester: Wiley; 2006. 10.1002/14651858.CD001500.pub2.

[18] Cardozo L, Lose G, McClish D, Versi E, de Koning GH (2001). A systematic review of estrogens for recurrent urinary tract infections: third report of the hormones and urogenital therapy (HUT) committee. Int Urogynecol J Pelvic Floor Dysfunct.

[19] Cardozo L, Benness C, Abbott D (1998). Low dose oestrogen prophylaxis for recurrent urinary tract infections in elderly women. BJOG An Int J Obstet Gynaecol.

[20] Brown M, Jones S (2005). Hyaluronic acid: a unique topical vehicle for the localized delivery of drugs to the skin. J Eur Acad Dermatol Venereol.

[21] Nusgens B-V (2010). Acide hyaluronique et matrice extracellulaire: une molécule primitive ?. Ann Dermatol Venereol.

[22] Ekin M, Yaşar L, Savan K, Temur M, Uhri M, Gencer I (2011). The comparison of hyaluronic acid vaginal tablets with estradiol vaginal tablets in the treatment of atrophic vaginitis: a randomized controlled trial. Arch Gynecol Obstet.

[23] Le Donne M, Caruso C, Mancuso A, Costa G, Iemmo R, Pizzimenti G (2011). The effect of vaginally administered genistein in comparison with hyaluronic acid on atrophic epithelium in postmenopause. Arch Gynecol Obstet.

[24] Serati M, Bogani G, Di Dedda MC, Braghiroli A, Uccella S, Cromi A (2015). A comparison between vaginal estrogen and vaginal hyaluronic for the treatment of dyspareunia in women using hormonal contraceptive. Eur J Obstet Gynecol Reprod Biol.

[25] Chen J, Geng L, Song X, Li H, Giordan N, Liao Q (2013). Evaluation of the efficacy and safety of hyaluronic acid vaginal gel to ease vaginal dryness: a multicenter, randomized, controlled, open-label, parallel-group. Clin Trial J Sex Med.

[26] Wylomanski S, Bouquin R, Philippe H-J, Poulin Y, Hanf M, Dréno B (2014). Psychometric properties of the French Female Sexual Function Index (FSFI). Qual Life Res.

[27] Bachmann G (1995). Urogenital ageing: an old problem newly recognized. Maturitas.

[28] Nugent RP, Krohn MA, Hillier SL (1991). Reliability of diagnosing bacterial vaginosis is improved by a standardized method of gram stain interpretation. J Clin Microbiol.

[29] Dahn A, Saunders S, Hammond J-A, Carter D, Kirjavainen P, Anukam K (2008). Effect of bacterial vaginosis, Lactobacillus and Premarin estrogen replacement therapy on vaginal gene expression changes. Microbes Infect.

[30] Yalcin I, Bump RC (2003). Validation of two global impression questionnaires for incontinence. Am J Obstet Gynecol.

[31] Quan T, Wang F, Shao Y, Rittié L, Xia W, Orringer JS (2013). Enhancing structural support of the dermal microenvironment activates fibroblasts, endothelial cells, and keratinocytes in aged human skin in vivo. J Invest Dermatol.

[32] Turlier V, Delalleau A, Casas C, Rouquier A, Bianchi P, Alvarez S (2013). Association between collagen production and mechanical stretching in dermal extracellular matrix: In vivo effect of cross-linked hyaluronic acid filler. A randomised, placebo-controlled study. J Dermatol Sci.

