# Supplementary material for: Evaluation of the effect of multipoint intra-mucosal vaginal injection of a specific cross-linked hyaluronic acid for vulvovaginal atrophy: a prospective bi-centric pilot study
Source: BMC Womens Health. 2021 Aug 28;21:322. doi: 10.1186/s12905-021-01435-w (PMC8403403; doi:10.1186/s12905-021-01435-w)
Supplement: Supplementary file 1 — Additional file 1. A poster summary of an unpublished study undertaken for DESIRIAL regulatory approvals. [file 12905_2021_1435_MOESM1_ESM.pdf]

Correction of female genital deficiencies through a new specific product range of hyaluronic acid gels.

N. Berreni, *MD*[1]; P. Marès, MD[1,2]; N. Tan, *Pharm.D*[3]; and D. Couchourel, *Ph.D; MBA*[1,3]

1: Genital Restoration and Innovation Research Group, Paris, France
2: Gynecology Department, Caremeau Hospital, Nimes, France
3: Laboratoires VIVACY, Archamps, France

INTRODUCTION:

Vulvo-vaginal atrophy (VVA) and labia majora atrophy are two ordinary groups of pathologies among women. More precisely, VVA commonly affects postmenopausal women, with prevalence estimates ranging from 10% to 50% *(Dennerstein L et al, 2000; Stenberg A et al, 1996; Chim H et al, 2002)*. Common symptoms include vaginal dryness, itching, irritation, and dyspareunia. The etiology of VVA among postmenopausal women is most commonly explained by the decrease in circulating estrogen associated with the menopausal transition, which has an adverse effect on skin (and mucosal) collagen and elasticity (*Bachmann et al, 2000; Johnston S et al, 2004; Keil K, 2002*).

The vaginal epithelium is a stratified squamous epithelium, which until menopause is moist and thick with rugae. At menopause, with declining levels of estrogen**, the vaginal epithelium thins**. Fewer epithelial cells result in less exfoliation of cells into the vagina. As epithelial cells exfoliate and die, they release glycogen, which is hydrolyzed to glucose. Glucose, in turn, is broken down into lactic acid by the action of lactobacillus, a normal vaginal commensal organism. Without this cascade, the pH in the vagina rises, resulting in a loss of lactobacilli and an overgrowth of other bacteria. **After menopause, the elasticity of the vagina is reduced and connective tissue quality is modified**. Regarding the hyaluronic acid (HA) content, the major age-related change is the increasing avidity of HA with tissue structures with the concomitant loss of HA extractability (Stern R et al, 2008). All of the above age related phenomena contribute to the apparent **dehydration, atrophy and loss of elasticity that characterizes aged tissues** *(Papakonstantinou E et al, 2012*). The initial symptom is often lack of lubrication during intercourse. Eventually, persistent vaginal dryness may occur. **Thinning of the epithelial lining** may also cause pruritus, soreness, and a stinging pain in the vaginal and vulvar area, which, in turn, may further contribute to dyspareunia *(MacBride et al, 2010*). This potential mechanism has leaded Laboratoires VIVACY to develop a new highly hygroscopic and easy to inject HA gel to replenish the ageing mucosae with qualitative high molecular weight HA molecules. The result of this work is a new product called Desirial.

The labia majora are also affected by the normal process of aging; time and gravity cause loss of dermal collagen and tissues elasticity. These effects lead to a **loss of tone and volume in the labia majora** whilst the labia minora increase in size causing discordance between the minor and major labia and an unattractive 'aged' appearance. Women frequently request 'vaginal rejuvenation' and in select patients, labia majora augmentation may offer significant aesthetic results for this particular portion of the vulva. However, very little is described in the literature regarding labia majora augmentation. To date, **the principal technique is based on fat auto-grafting** which needs surgical procedure. Hence, Laboratoires VIVACY proposes Desirial plus, a new injectable HA, which is specifically designed to adapt its rheological properties to the very specific mechanical environment of the female genital sphere. The procedure is simple and feasible in the physician office but to be efficient, stability and good integration in the tissues are crucial to cope with high mechanical stress of this zone.

MATERIAL & METHODS:

A population of 95 women was recruited on the basis of the presence of two main indications: VVA and/or labia majora fat atrophy. More precisely, 52 patients were treated with Desirial for VVA, and 35 patients were treated with Desirial plus for labia majora fat atrophy:

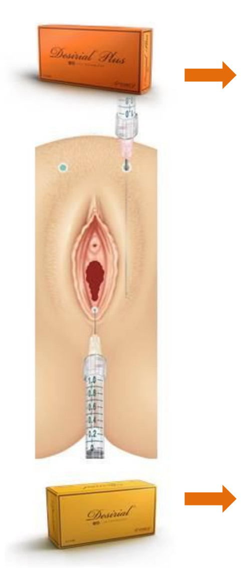

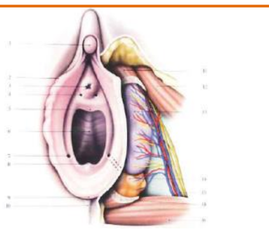

Injection plan for Desirial plus. The product is placed using a **cannula**, in the fat layer, between the muscular dartos and the labia majora erectile corpus (embedded in the Sappey fibrous bag)

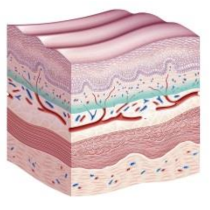

Injection plan for Desirial. The product is placed using a **sharp needle** just under the mucosae, at the interface with the spongy tissue layer (highly vascularized)

Clinical follow-up of the patients was performed during **12 months**. Several markers have been recorded:

1) Functional

a) Vulvo-vaginal pH
b) VV mucosae inflammation
c) VV mucosae dryness
d) Dyspareunia
e) Global life improvement (GLIS)

2) Aesthetic:

a) Labia Majora volume
b) Itching
c) Chafing
d) Global life improvement (GLIS) and global aesthetic improvement (GAIS)

RESULTS:

1) Functional

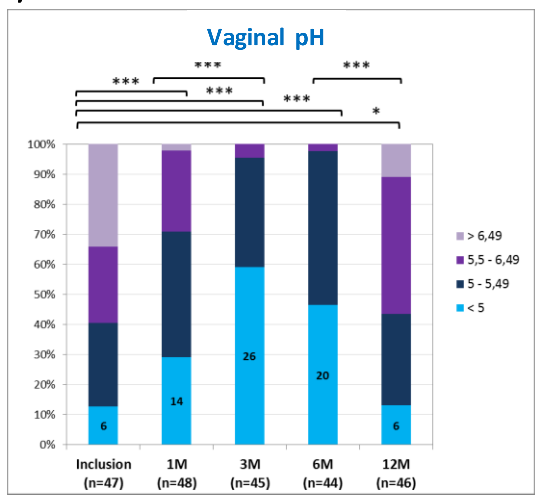

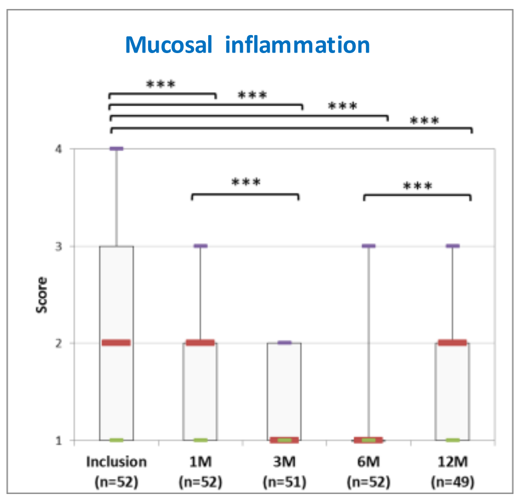

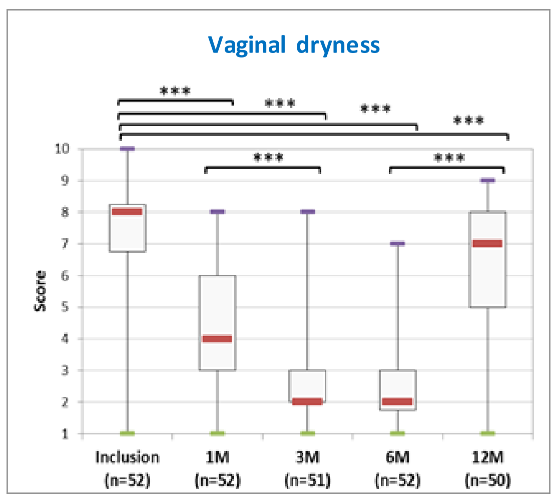

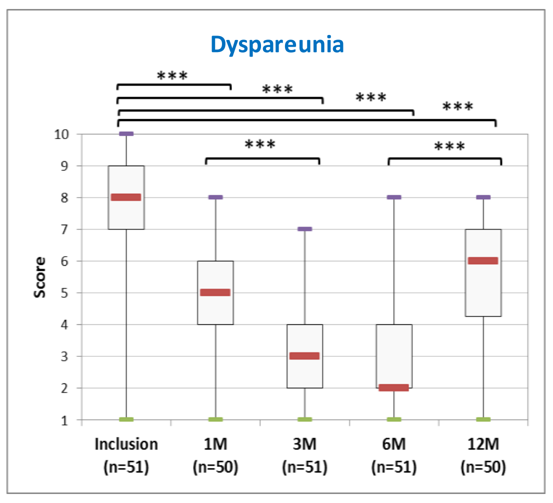

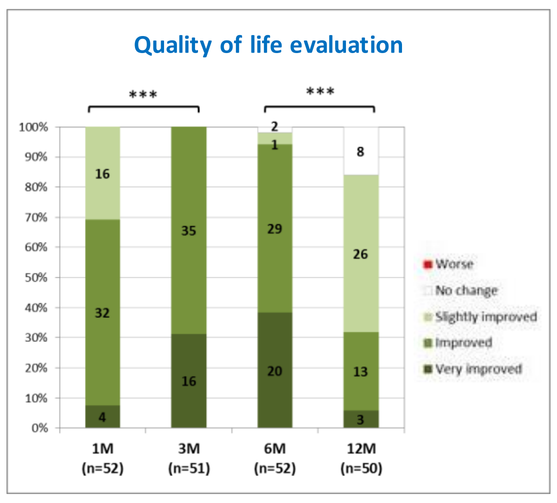

2) Aesthetic

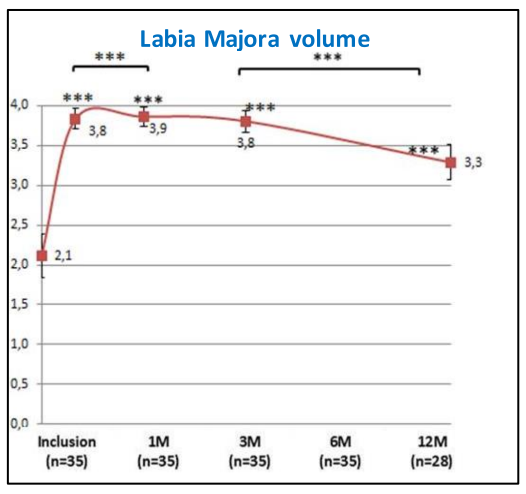

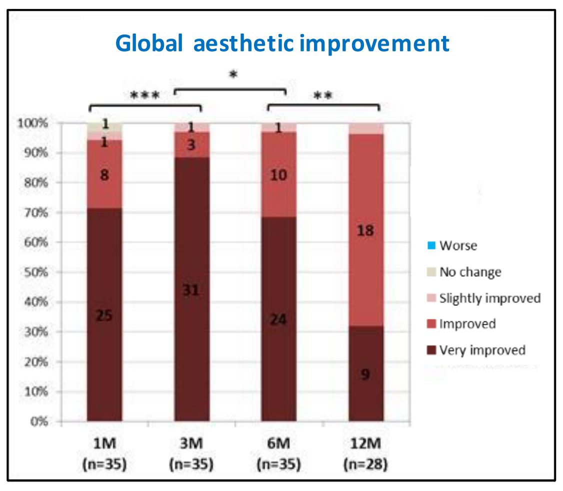

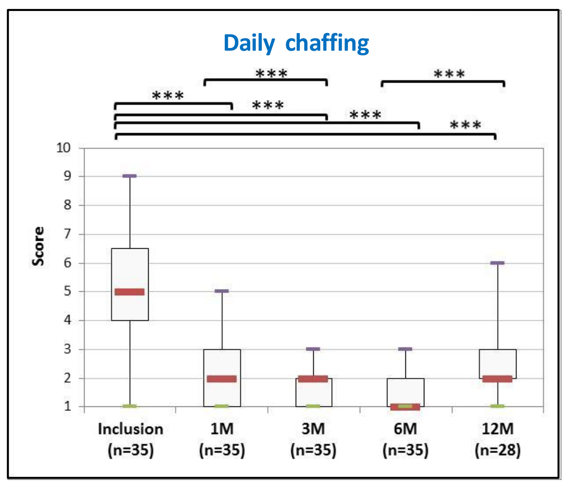

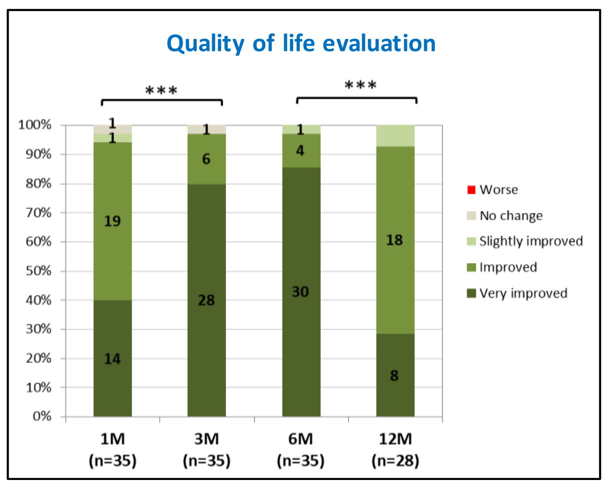

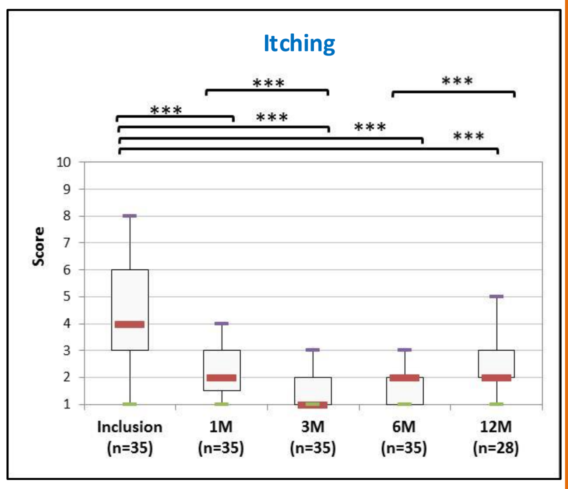

CONCLUSION

On the functional aspects, this work demonstrates that sub-mucosal Desirial injections could improve the comfort of VVA suffering patients. More precisely, vaginal pH is significantly decreased after the treatment. Moreover, this improvement remains significant 6 months post-injection. As we discussed above, the beneficial evolution of this objective clinical marker could represent one of the causes of the more subjective results showed in this report. Indeed, mucosal inflammation, vaginal dryness (physician evaluation) as well as dyspareunia (patient evaluation) are significantly improved. As a consequence, GLIS is clearly positively impacted by the treatment. These promising results need to be put in perspective with the already existing solutions for those patients. Beside hormonal supplementation based treatments, two types of topical treatments can be considered. First, vaginal hydration gels usually contain glycerin, palm oil glycerin, paraffin wax, and hydrophilic compounds. They can trigger positive variations of the vaginal maturation index and increase of vaginal secretions. In addition, it has been demonstrated that those products could improve vaginal itching, chafing and also dyspareunia. Secondly, vaginal lubricant gels are also available. They are usually used to decrease irritation and chafing during sexual intercourse. However, it has been demonstrated that OTC treatments compliance is poor: messiness, lack of symptoms relief, inconvenience, lack of sexual spontaneity have been identified as the main causes *(Wysocki et al, 2014)*. Thus, Desirial submucosal treatment appears to be an highly promising option which can be prescribed in association with above mentioned classical options. Regarding safety aspects, the most frequent recorded adverse events were described as hematoma, edema and redness. Resolutions were quick and no specific treatments were needed.

Desirial Plus is dedicated to labia majora reconstruction. 35 patients were treated for weak to medium fat atrophy. Results show that volume restauration was efficient and still significantly present 6 months after treatment. Moreover, this result is confirmed by the GAIS evolution: there is a trend showing that patients were still satisfied of the results 12 months after treatment. Logically, these results should be confirmed when the 12-month follow-up of all the included patients will be completed. Interestingly, it is also evident that functional improvement are demonstrated even if the initial demand was for aesthetic purpose.

In conclusion, Desirial and Desirial Plus represent new treatment options for VVA and labia majora atrophy. This work needs to be confirmed with a placebo design study for an evidence based medicine demonstration.

REFERENCES

- Chim H, Tan B, Ang C, Chew E, Chong Y, Saw S. The prevalence of menopausal symptoms in a community in Singapore. Maturitas 2002; 41:275-282
- Bachmann G, Nevadunsky N. Diagnosis and treatment of atrophic vaginitis. Am Fam Physician 200;61:3090-3096.
- Dennerstein L, Dudley E, Hopper J, Guthrie J, Burger H. Obstet Gynecol 2000; 96:351-358
- Johnston S, Farrell S. J Obstet Gynaecol Can 2004;26:503-508
- Keil K. Curr Womens Health Rep 2002;2:305-311
- Mac Bride MB, Rhodes DJ, Shuster LT. Mayo Clin Proc. 2010 Jan;85(1):87-94
- Papakonstantinou E, Roth M, Karakiulakis G. Dermatoendocrinol. 2012 Jul 1;4(3):253-8
- Stenberg A, Heimer G, Ulmsten U, Snattingus S. Maturitas 1996;24:31-36
- Wysocki S et al, Clin Med Insights Reprod Health. 2014 Jun 8;8:23-30
